# Low dose pramipexole is neuroprotective in the MPTP mouse model of Parkinson's disease, and downregulates the dopamine transporter via the D_3 _receptor

**DOI:** 10.1186/1741-7007-2-22

**Published:** 2004-10-11

**Authors:** Jeffrey N Joyce, Cheryl Woolsey, Han Ryoo, Sabine Borwege, Diane Hagner

**Affiliations:** 1 Thomas H. Christopher Center for Parkinson's Disease Research, Sun Health Research Institute, 10515 West Santa Fe Dr., Sun City, AZ, 85352, USA

## Abstract

**Background:**

Our aim was to determine if pramipexole, a D_3 _preferring agonist, effectively reduced dopamine neuron and fiber loss in the 1-methyl-4-phenyl-1,2,3,6-tetrahydropyridine (MPTP) mouse model when given at intraperitoneal doses corresponding to clinical doses. We also determined whether subchronic treatment with pramipexole regulates dopamine transporter function, thereby reducing intracellular transport of the active metabolite of MPTP, 1-methyl-4-phenylpyridinium (MPP+).

**Methods:**

Ten 12-month old C57BL/6 mice were treated with MPTP (or saline) twice per day at 20 mg/kg s.c. (4 injections over 48 h). Mice were pretreated for 3 days and during the 2-day MPTP regimen with pramipexole (0.1 mg/kg/day) or saline. Stereological quantification of dopamine neuron number and optical density measurement of dopamine fiber loss were carried out at 1 week after treatment, using immunostaining for dopamine transporter (DAT) and tyrosine hydroxylase (TH). Additional wild-type (WT) and D_3 _receptor knockout (KO) mice were treated for 5 days with pramipexole (0.1 mg/kg/day) or vehicle. The kinetics of [^3^H]MPP+ and [^3^H]DA uptake (*V*_max _and *K*_m_) were determined 24 h later; and at 24 h and 14 days dopamine transporter density was measured by quantitative autoradiography.

**Results:**

Pramipexole treatment completely antagonized the neurotoxic effects of MPTP, as measured by substantia nigra and ventral tegmental area TH-immunoreactive cell counts. MPTP- induced loss of striatal innervation, as measured by DAT-immunoreactivity, was partially prevented by pramipexole, but not with regard to TH-IR. Pramipexole also reduced DAT- immunoreactivity in non-MPTP treated mice. Subchronic treatment with pramipexole lowered the *V*_max _for [^3^H]DA and [^3^H]MPP^+ ^uptake into striatal synaptosomes of WT mice. Pramipexole treatment lowered *V*_max _in WT but not D_3 _KO mice; however, D_3 _KO mice had lower *V*_max _for [^3^H]DA uptake. There was no change in DAT number in WT with pramipexole treatment or D_3 _KO mice at 24 h post-treatment, but there was a reduction in WT-pramipexole treated and not in D_3 _KO mice at 14 days post-treatment.

**Conclusion:**

These results suggest that protection occurs at clinically suitable doses of pramipexole. Protection could be due to a reduced amount of MPP^+ ^taken up into DA terminals via DAT. D_3 _receptor plays an important role in this regulation of transporter uptake and availability.

## Background

An interesting development in the use of dopamine (DA) agonists for treatment of Parkinson's disease (PD) is that some of them have proven to be neuroprotective in animal models of PD. Antiparkinsonian agents that are direct DA agonists, such as apomorphine [1], bromocriptine [2], and pramipexole [3], are neuroprotective against 1-methyl-4-phenyl-1,2,3,6-tetrahydropyridine (MPTP)-induced damage to the DA system in mice. Administration of MPTP, which is converted to 1-methyl-4-phenylpyridinium (MPP+) and intracellularly transported into DAergic neurons [4], provides a good model for studying neuroprotection in PD. MPTP produces Parkinsonism in humans and in subhuman species through selective loss of DAergic neurons of the substantia nigra (SN) [5,6], and a number of related compounds to MPTP also produce nigral cell loss in primates [7]. MPTP causes apoptosis associated with PD [8-10] ;MPTP produces progressive cell death in humans for decades after the initial insult [11]. Hence, drugs that reduce the neurotoxicity of compounds like MPTP may be neuroprotective in PD. In fact, it is now hypothesized that direct DA agonists may slow the loss of DAergic terminal function upon long-term administration to PD patients [12-15].

Dopaminergic neurons are tonically inhibited by dendritic and terminal autoreceptors, operating in interaction with DA transporters (DAT) and pharmacologically of the D_2 _receptor subtype [16-19]. However, Zapata et al [20] have reported that the D_3 _preferring agonist (+)-PD 128907 regulates extracellular DA levels via interactions with D_3 _autoreceptors. If D_3 _preferring agonists are potent autoreceptor agonists, then hypothetically long-term changes in expression of DAT or the functional properties of DAT might occur following subchronic treatment. Since intracellular accumulation of MPP+ following systemic injection of MPTP requires DAT [4], then when DAT is downregulated by D_3 _preferring agonists, this could result in lower intracellular accumulation of MPP+ and reduced neurotoxicity to MPTP.

The D_3 _receptor preferring agonists, pramipexole and ropinirole, are the most potent of the DA agonists affording neuroprotection at 1 mg/kg for pramipexole against MPTP-induced neurodegeneration [3,21] and at 2 mg/kg for ropinirole against 6-OHDA lesions in rats [22]. Doses 10–30 times higher of DA agonists with low D_3 _receptor affinity such as apomorphine [1] and bromocriptine [2,23] are needed against MPTP-induced neurodegeneration. Because neuroprotection by pramipexole is most evident with concurrent treatment with MPTP and not with post-MPTP treatment [24], i.e. when autoreceptor contributions should be most pronounced, regulation of DAT may be important. In addition, while the lowest effective dose reported is 1.0 mg/kg for mice, this is significantly greater than a clinically relevant dose in humans (1.5 mg t.i.d., p.o.[25]). Based on information from Pharmacia Corporation, equivalent plasma levels obtained with 1.5 mg t.i.d., p.o. in humans could be produced with 0.1 to 0.5 mg/kg in the mice. We tested whether 0.1 mg/kg pramipexole would be neuroprotective in aging mice against MPTP-induced neurodegeneration to the DA system, and if this effect could be due to regulation of DAT function.

## Results

### Neurohistopathology

**Figure 1:**
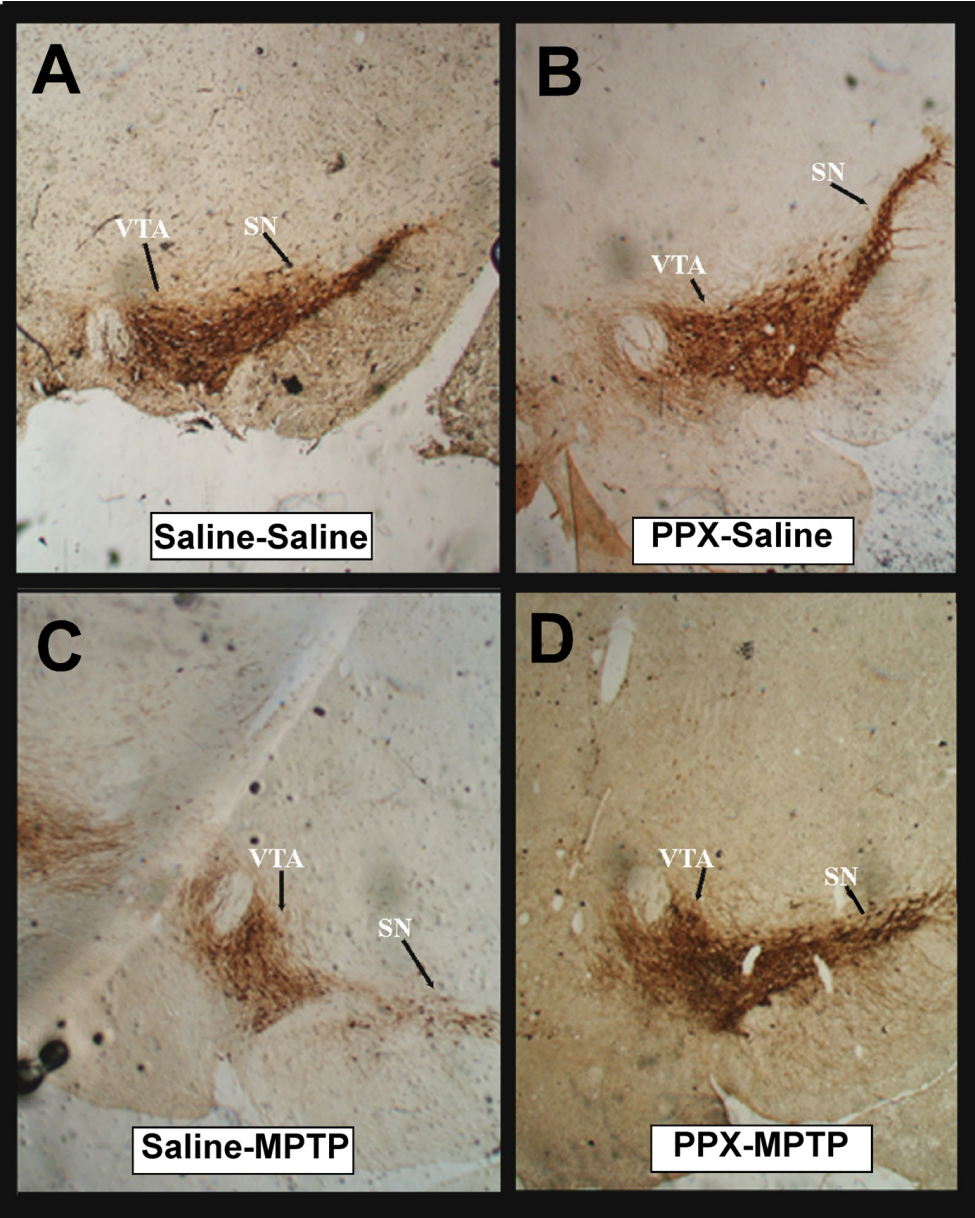
**Impact of pramipexole on MPTP-induced loss of TH-IR neurons. **Low-power photomicrograph (20x) of the substantia nigra and ventral tegmental area of tissue sections stained for TH-IR from individual cases of 4 groups of mice: (A) Veh-Veh, (B) pramipexole -Veh, (C) Veh-MPTP (25 mg/kg sc., 4 times in 2 days at 8 h intervals), (D) pramipexole-MPTP. Note the marked depletion of TH-IR from the substantia nigra and considerably lesser impact in the ventral tegmental area following MPTP. Pramipexole pretreatment regimen provided complete protection (see D) from MPTP. Abbreviations: SNpc, substantia nigra pars compacta; VTA, ventral tegmental area.

**Figure 2:**
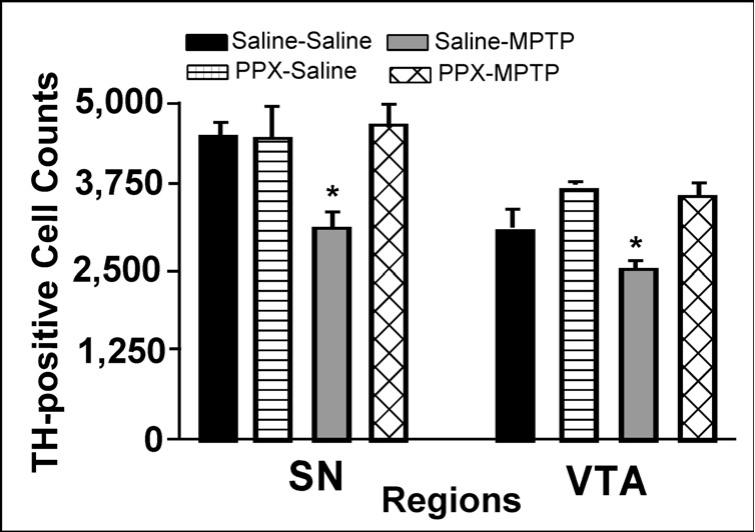
**Histogram of pramipexole's neuroprotective effects against MPTP induced cell loss. **Effects of subchronic treatment with pramipexole on total TH-IR cell counts in the substantia nigra and ventral tegmental area of mice treated with or without MPTP. Pramipexole were administered 3 days prior to and during the 2-day treatment with MPTP. Animals survived for 7 days after the last MPTP injection. Data are mean ± standard error of the mean from 4–5 samples per group. * P < 0.05 vs all other groups

Male C57BL/6 mice of 8–10 months of age were pretreated with saline or pramipexole (0.1 mg/kg/day) followed by MPTP. At the end of the 7-day recovery period following the last injection of MPTP or vehicle were assessed for the degree of toxicity to the dopamine system by MPTP. MPTP produced a marked loss of tyrosine hydroxylase-immunoreactive (TH-IR) neurons in the substantia nigra (SN), but had less impact in the ventral tegmental area (VTA) (Figs 1 and 2), Unbiased stereological quantification of the number of Nissl-stained and TH-IR neurons in the SNpc and VTA was made in the midbrains of the treated groups. MPTP produced a 31% loss of TH-IR neurons in the SN and 17% loss in the VTA. Pramipexole administered once a day for 5 days (i.e. 3 days prior to and during the 2-day vehicle treatment) did not alter the total number of TH-IR neurons in the SN or VTA. Pramipexole administered for 3 days prior to and during the 2-day administration of MPTP completely prevented TH-IR neuron loss in the SN and VTA of MPTP treated mice. To confirm that TH-IR neurons were dead and not simply exhibiting reduced TH-IR, neurons in Nissl stained sections were counted. The results confirmed the data that D_3-_preferring agonists can protect against MPTP in vivo as well as against MPP+ in vitro [26,27].

**Figure 3:**
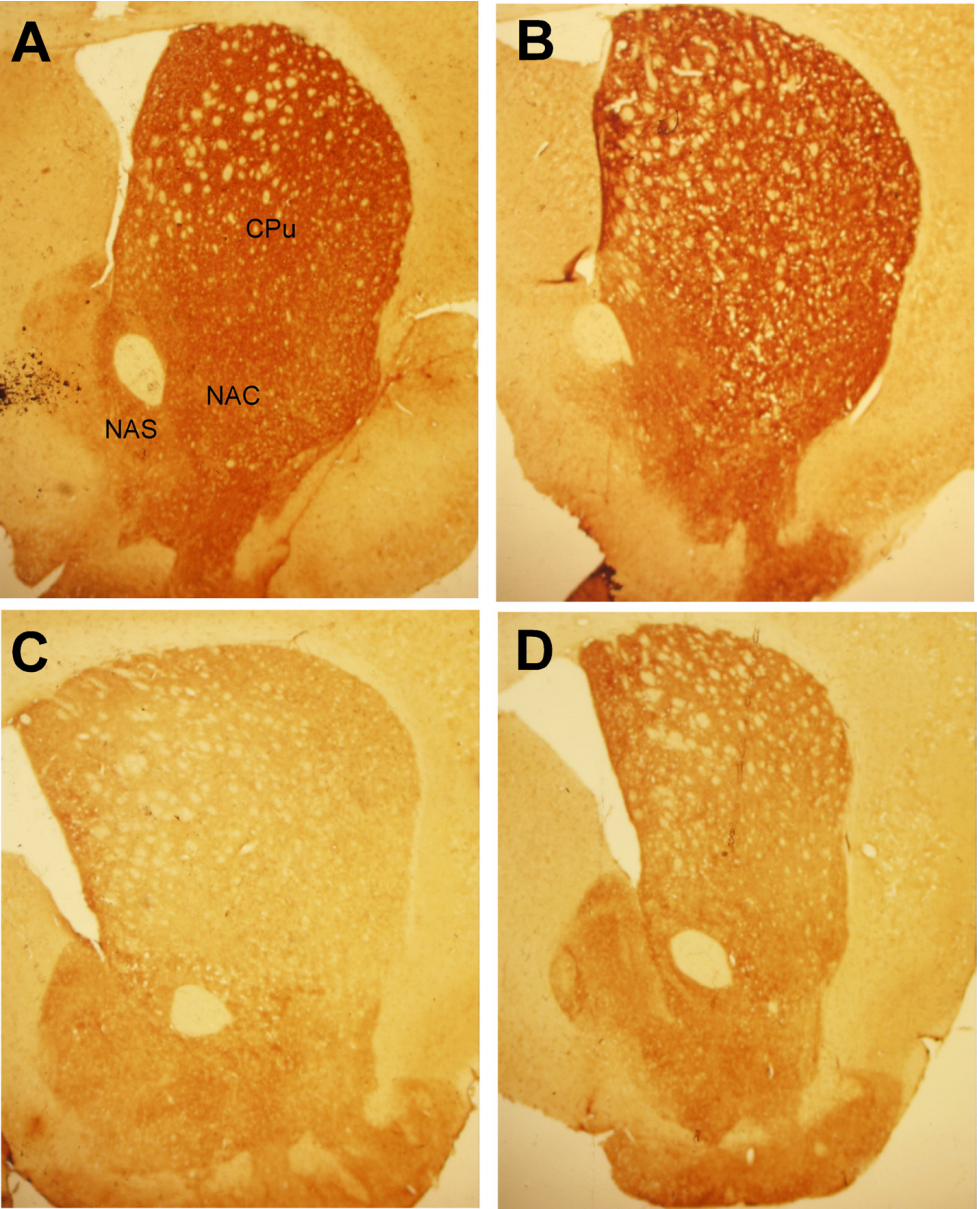
**Impact of pramipexole on MPTP-induced loss of DAT-IR fibers. **Low-power photomicrograph (20x) of the caudate-putamen and nucleus accumbens of tissue sections processed for DAT-IR from individual cases of 4 groups of mice: (A) Saline-Saline, (B) pramipexole – Saline, (C) Saline -MPTP (20 mg/kg sc., 4 times in 2 days at 8 h intervals), (D) pramipexole-MPTP. Note the marked depletion of DAT-IR from the caudate-putamen (C) and apparent protection by pramipexole (D). Abbreviation: CPu, caudate-putamen; NAS, nucleus accumbens shell; NAC, nucleus accumbens core.

**Figure 4:**
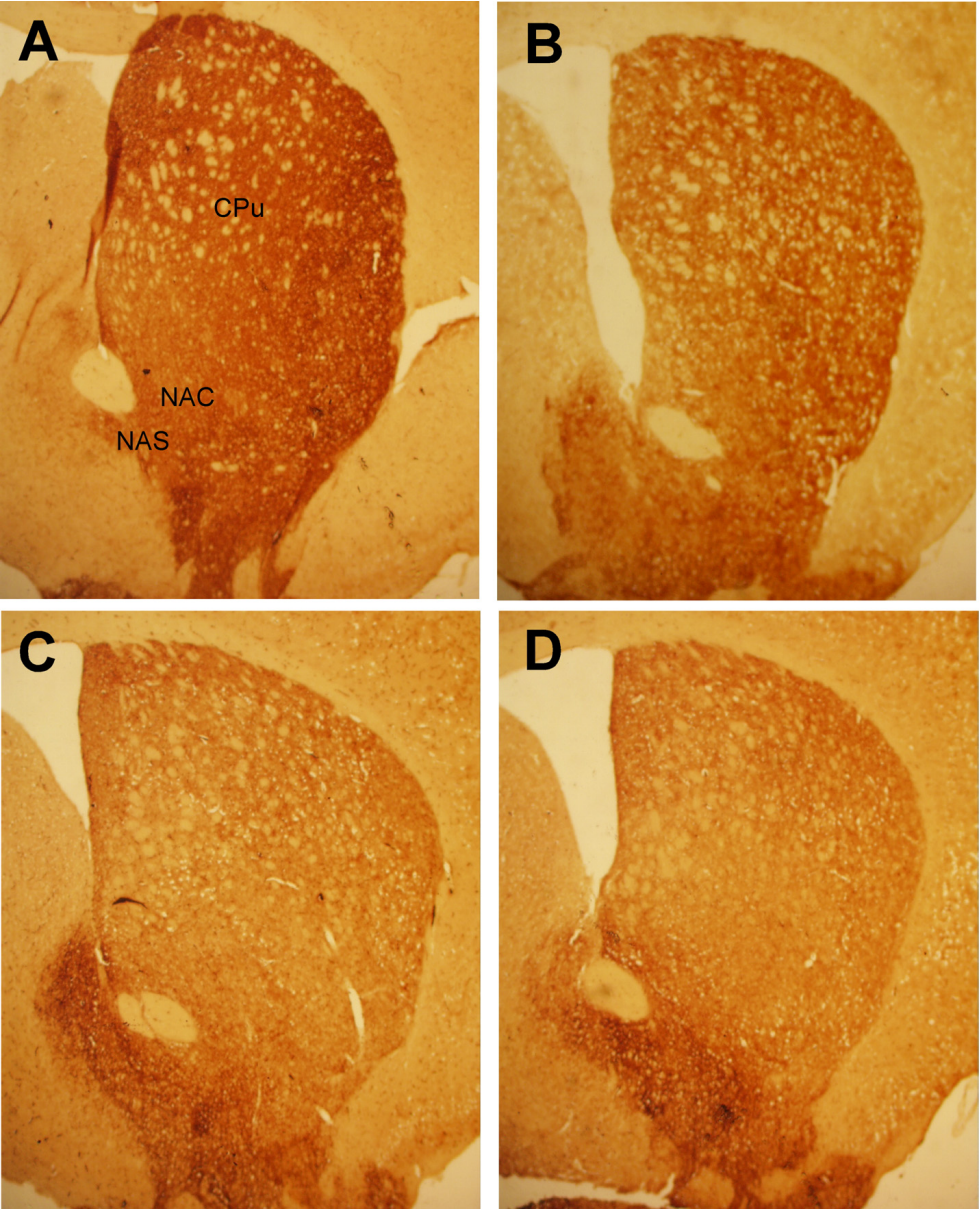
**Impact of pramipexole on MPTP-induced loss of TH-IR fibers. **Low-power photomicrograph (20x) of the caudate-putamen and nucleus accumbens of tissue sections processed for TH-IR from individual cases of 4 groups of mice: (A) Saline-Saline, (B) pramipexole – Saline, (C) Saline -MPTP (20 mg/kg sc., 4 times in 2 days at 8 h intervals), (D) pramipexole-MPTP. Note the marked depletion of TH-IR from the caudate-putamen (C) and apparent protection by pramipexole (D). Abbreviation: PPX, pramipexole.

**Figure 5:**
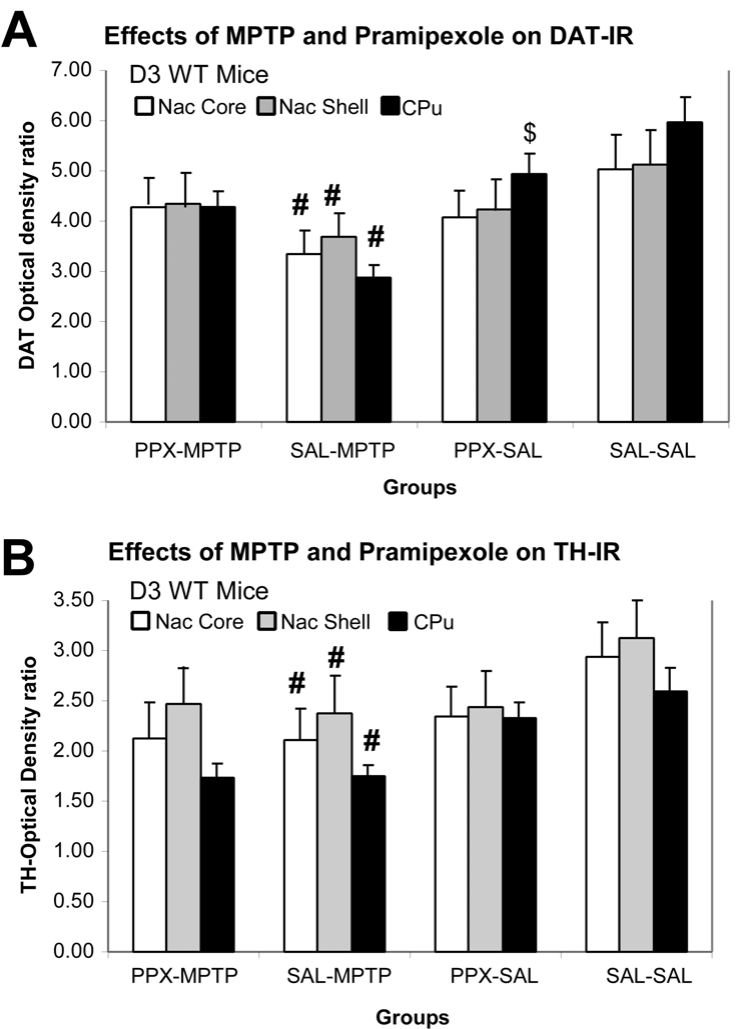
**Histogram of pramipexole's neuroprotective effects against MPTP induced fiber loss. **Effects of subchronic treatment with pramipexole on DAT-IR (A) and TH-IR (B) optical density in the caudate-putamen, nucleus accumbens core and shell of mice treated with or without MPTP. Animals survived for 7 days after the last MPTP injection. Data are mean ± standard error of the mean from 4–5 samples per group. # for P < 0.01, all groups vs SAL + MPTP group; $ for P < 0.01, for SAL-PPX vs SAL-SAL. Abbreviations: CPU, caudate-putamen; Nac Core, nucleus accumbens core; Nac Shell, nucleus accumbens shell; PPX-MPTP, pramipexole + MPTP treated group; SAL-MPTP, saline + MPTP treated group; PPX-SAL, pramipexole + saline treated group; SAL-SAL, saline + saline treated group.

Visualization of DA fibers with dopamine transporter immunoreactivity, DAT-IR (Fig 3), and TH-IR (Fig 4) demonstrated uniform staining of the caudate-putamen (CPu) and nucleus accumbens (Nac) in the saline/saline cases. In saline pretreated mice, MPTP reduced DAT-IR by 52% in the CPu (Figs 3 and 5A) and had a smaller but significant impact on DAT-IR labeling of DA fibers in the Nac. DAT-IR in the CPu in the pramipexole plus vehicle (PPX-SAL)-treated mice was reduced by 17%. Furthermore, in pramipexole and MPTP treated mice, a significant attenuation of the impact of MPTP in the CPu (-27% vs -52% loss) and Nac of MPTP treated mice was seen.

In contrast to DAT-IR, levels of TH-IR (Figs 4 and 5B) was not significantly reduced (~11%) by pramipexole in PPX-SAL group. MPTP produced a significant (34%) loss of TH-IR labeling of DA fibers in the CPu of WT mice and to a lesser degree in the Nac SAL-MPTP group. Pramipexole did not significantly attenuate the effects of MPTP on TH-IR labeling of DA fibers in the CPu of WT mice (Fig 5B).

### [^3^H]MPP+ and [^3^H]dopamine uptake in mouse striatal postnuclear preparations

**Figure 6:**
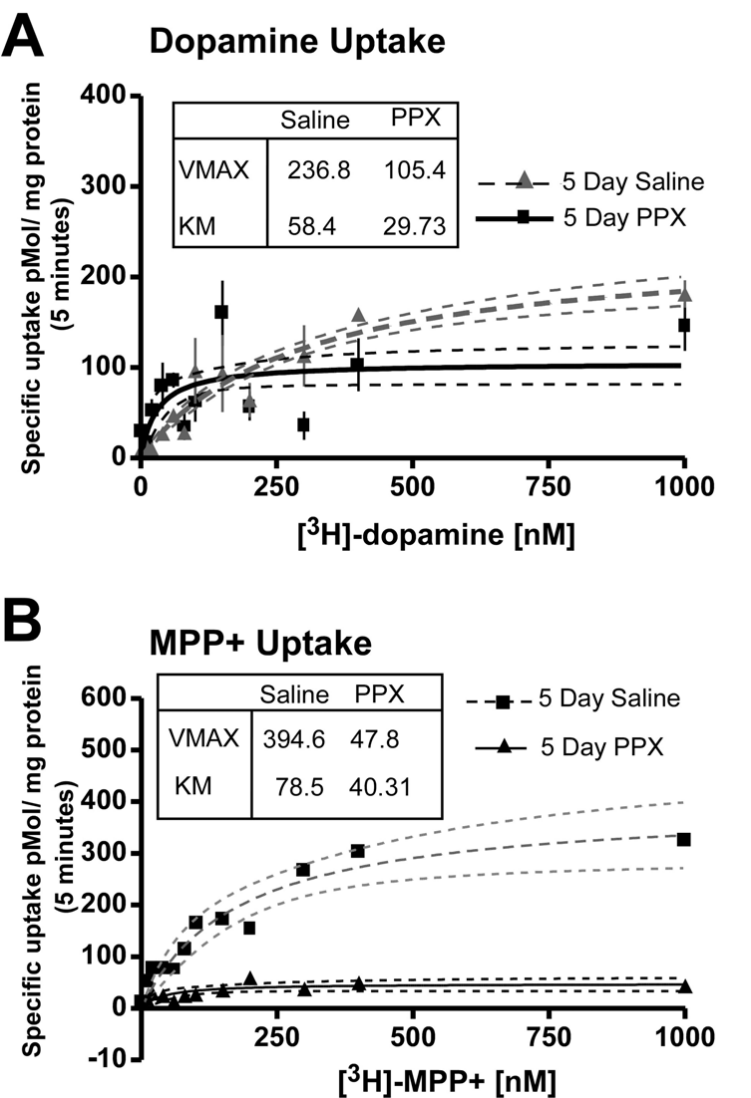
**Effects of pramipexole on the kinetics of [^3^H]DA and [^3^H]MPP+ uptake. **Effects of subchronic treatment pramipexole or vehicle (saline) on [^3^H]DA uptake (A) and [^3^H]MPP+ uptake (B) in striatal synaptosomes from treated mice. Group data are plotted with the mean ± standard error of the mean from 6 samples per group. The dashed line for each of the means represents the calculated 95% confidence interval. The *V*_max _and *K*_m _were calculated for each group and paired comparisons by t-test revealed significant differences.

Since pramipexole treatment in non-MPTP treated mice altered levels of DAT-IR, it was important to identify if pramipexole treatment altered the kinetics of [^3^H]DA and [^3^H]MPP+ uptake through the DAT. Kinetics of [^3^H]DA uptake in mouse striata demonstrated a higher *V*_max _but a similar *K*_m _to that reported for rat fresh striatal postnuclear preparation [28]. Uptake was sodium dependent and the *K*_m _was similar due to inclusion of COMT inhibitor in the preparations [29]. C57BL/6 mice treated with pramipexole once a day for 5 days exhibited significant differences from saline treated mice (Fig 6). The *V*_max _(P = 0.0008) and the *K*_m _(P = 0.016) for [^3^H]DA uptake was significantly decreased in pramipexole treated mice. The *V*_max _(P < 0.001) and the *K*_m _(P = 0.001) for [^3^H]MPP^+ ^uptake were also significantly decreased in pramipexole treated mice.

To determine if the reduction in *V*_max _and the *K*_m _for [^3^H]DA uptake by subchronic treatment with pramipexole was due to the D_3 _receptor, the values for *V*_max _and the *K*_m _for [^3^H]DA uptake was measured in WT (C57BL/6) and D_3 _KO littermate mice (bred according to our previous methods [30]), treated with vehicle or pramipexole for 5 days. ANOVA showed that there were group differences for *V*_max _values (F = 72.41, P < 0.0001) and for *K*_m _values (F = 9.78, P = 0.0007). Synaptosomes prepared from D_3 _KO mice had significantly lower *V*_max _(P = 0.001, by 59%) and *K*_m _(P = 0.01, by 69 %), values for [^3^H]DA uptake than WT mice (Table 1). WT mice treated with pramipexole significantly lowered *V*_max _(P = 0.001, by 65%) and *K*_m _(P = 0.05, by 50%) values for [^3^H]DA uptake compared with WT mice treated with saline. D_3 _KO mice treated with pramipexole had significantly lower *V*_max _(P = 0.001, by 57%) and *K*_m _(P = 0.051, by 77%) values for [^3^H]DA uptake than WT mice treated with saline, but D_3 _KO mice treated with pramipexole were not different from D_3 _KO mice treated with vehicle. WT mice treated with pramipexole were also not different from D_3 _KO mice treated with pramipexole.

### Dopamine transporter (DAT) autoradiography

**Figure 7:**
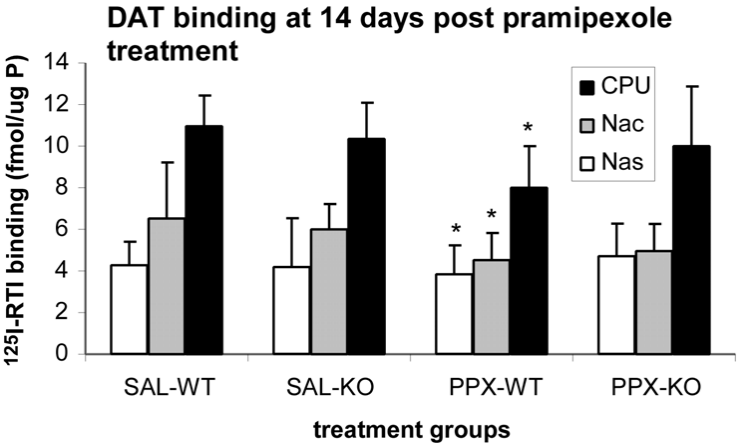
**Effects of pramipexole on DAT density at 14 days post-treatment. **Effects of subchronic treatment with pramipexole on density of [^125^I] RTI-55 labeled sites by autoradiography. Animals survived for 14 days after the last pramipexole injection. Data are mean ± standard deviation of the mean from 4–5 samples per group. * for P < 0.01, PPX-WT vs all groups. Abbreviations: CPU, caudate-putamen; Nac Core, nucleus accumbens core; Nac Shell, nucleus accumbens shell; PPX-KO, pramipexole treated D_3 _knockout mice; PPX-WT, pramipexole treated WT mice; SAL-KO, saline treated D_3 _knockout mice; SAL-WT, saline treated WT mice.

To determine if the reduction in *V*_max _and the *K*_m _for [^3^H]DA uptake by subchronic treatment with pramipexole at 24 h post-treatment was due to the D_3 _receptor regulation of the number of DAT sites, the density of DAT sites was measured in WT and D_3 _KO mice treated with vehicle or pramipexole for 5 days. ANOVA showed that there were no group differences (Table 1) at 24 h post-treatment, but by 14 days there was a significant reduction of DAT sites in WT mice treated with pramipexole (P < 0.01), but not in D_3 _KO mice (Fig 7). WT mice treated with pramipexole exhibited a 27% reduction in the CPu, a 31% reduction of [^125^I]RTI binding to DAT in the Nac, and a 11% reduction in the Nas compared to WT mice treated with vehicle.

## Discussion

Antiparkinsonian agents that are direct DA agonists (e.g. apomorphine[1], bromocriptine [2,23], and pramipexole [3]) are almost completely neuroprotective against MPTP-induced loss of striatal DA in mice. MPTP administration to mice can produce varying degrees of effect on permanent damage to the nigrostriatal DA system depending on the schedule of MPTP administration and survival period after MPTP [31-36]. In addition, under some conditions dramatic recovery from the initial loss of striatal DA levels can occur without any intervention [33,34]. Therefore, studies employing measures of striatal DA levels as the sole criteria for impact of MPTP and protection by DA agonists do not offer convincing evidence of protection [1-3,23,37]. Three studies have employed both unbiased stereological counts of TH-IR neurons in the midbrain along with estimate of striatal DA content of animals administered MPTP [21,24] or intracerebroventricular 6-hydroxoydopamine [38] to produce permanent damage to the DA system with protection by pramipexole. In all 3 studies the impact of MPTP on striatal DA levels was reduced by 50% with pretreatment or concurrent plus post-MPTP treatment with pramipexole (1 to 3 mg/kg). Protection against midbrain TH-IR neurons was greater, even at 12 to 14 days post MPTP/6-OHDA treatment than against DA levels. In this study of a much lower dose of pramipexole (10 to 30 times lower) given 3 days prior to and during the 2-day MPTP treatment, at 1-week post-MPTP pramipexole treatment completely spared the substantial MPTP-induced loss of TH-IR neurons from the SNpc, but was less effective against the loss of DA fibers in the CPu (TH-IR and DAT-IR measures). Measurements of striatal DA [21,24] which show more protection by pramipexole than our estimates of DA fibers in the CPu (TH-IR and DAT-IR measures) may reflect upregulation of DA synthesis in the remaining fibers rather than protection against fiber loss. In addition, pramipexole treatment by itself reduced DAT-IR of DA fibers, suggesting that protection against MPTP might be greater than our immunocytochemical measures indicate.

The interesting observation that pramipexole down-regulated DAT-IR in saline treated mice suggests that DAT regulation plays a role in neuroprotective effects of pramipexole. D_3 _receptors are most concentrated in the brain on neurons in the nucleus accumbens, however DAergic neurons do express both D_2 _and D_3 _receptors [39,40], and both D_2 _and D_3 _receptors are functional autoreceptors [16-20,41,42]. Dopaminergic neurons are tonically inhibited by dendritic and terminal autoreceptors operating in interaction with DA transporters and pharmacologically of the D2 receptor type [16-19]. Zapata et al [20] have reported that the D_3 _preferring agonist (+)-PD 128907 does interact with D_3 _autoreceptors to regulate extracellular DA levels. Those data are consistent with acute treatment with D_2_/D_3 _agonists leading to increased *V*_max _[42,43], but less is known about subchronic treatment.

We observed that subchronic treatment with pramipexole reduced the *V*_max_, and *K*_m_, of [^3^H]DA and [^3^H]MPP+ uptake in mice at 24 h post-treatment. Interestingly, D_3 _receptor KO mice exhibited substantially lower *V*_max _and *K*_m _values of [^3^H]DA uptake than WT mice, and pramipexole did not further reduce *V*_max _and *K*_m _values in D_3 _receptor KO mice. However, the actual density of sites on the DA terminals was not reduced 24 h after pramipexole treatment in WT mice (or in D_3 _receptor KO mice), as determined by DAT autoradiography. The inability to modulate the number of DAT binding sites 12 h after termination of subchronic treatment with D_2_/D_3 _receptor agonists has previously been reported [44], suggesting that the initial alteration of *V*_max _and *K*_m _with pramipexole might be due to modification of the kinetics of DAT. However, pramipexole treatment reduced DAT-IR of DA fibers 7 days post-treatment, suggesting a reduction in DAT sites. Consistent with this, there was a reduction of [^125^I]RTI binding to DAT sites in pramipexole treated WT mice 14 days after pramipexole treatment, but not in D_3 _receptor KO mice. Thus, there might be both rapid and slower modifications in DAT function produced by D_3_/D_2 _agonist treatment, ultimately resulting in lower DAT number, and mediated by the D_3 _receptor.

It is known that DAT half-life in the striatum is decreased by D_2_/D_3 _receptor agonists, and increased by the dopamine D_2_/D_3 _receptor antagonist, but not by D1 agonists and antagonists [45]. Furthermore, the D2 agonist-induced change in DAT kinetics iss inhibited by the co-administration of an antagonist. The absence of DA receptors can also influence DAT function, as shown by dopamine D_2 _receptor-deficient mice, which exhibit decreased striatal DA uptake [19]. The present results can be compared with those reported by Saunders et al. [46] using hDAT-FLAG expressed in human embryonic kidney 293-EM4 cells, who showed by confocal microscopy and whole-cell current recordings that 2 μM *d*-amphetamine increased internalization of surface DAT within 1 h. Treatment with DA in HEK-hDAT cells also reduced *V*_max_, due to a diminished presence of DAT at the surface of synaptosomes [47]. Subchronic pramipexole might lead to redistribution DAT from the plasma membrane to endosomal compartments, and regulated, in part, by the D_3 _receptor. The initial change in *V*_max_, and *K*_m_, could be related to a more rapid turnover of DAT, and the longer-term reduction in Bmax to greater internalization and/or reduced synthesis. Thus, D_3 _preferring agonists might be potent autoreceptor agonists, and long-term changes in expression of the DAT or the functional properties of DAT could occur following subchronic treatment. This, in turn, could lead to reduced MPP^+ ^(and other neurotoxins) uptake into DA neurons, and reduced toxicity in animal models of PD. Ramirez and associates [37] reported that the neuroprotective effects of pramipexole against MPTP-induced DA loss in mice was attenuated by the selective D_3 _antagonist A-437203. Furthermore, the neuroprotective effect of a low dose of pramipexole was attenuated in D_3 _transgenic knockout mice and protection by pramipexole was not further attenuated by treatment with a D_3 _antagonist. These *in vivo *data support an important role for the D_3 _receptor in the neuroprotective effects of DA agonists, and our data suggest that this, in part, is due to reduced MPP^+ ^uptake into DA neurons.

## Conclusions

We have identified that subchronic treatment with a clinically relevant dose of pramipexole beginning before initiation of MPTP treatment affords neuroprotection against DA neuron loss and, to a lesser extent, DA fiber loss. This might involve down-regulation of DAT and reduced MPP+ uptake into DA fibers. Since intracellular accumulation of MPP+ following systemic injection of MPTP requires DAT [4], then if DAT function and/or number are reduced by D_3 _preferring agonists this could result in lower intracellular accumulation of MPP+ and reduced neurotoxicity to MPTP. The importance of knowing the targets of pramipexole, and other D_3 _preferring agonists, in neuroprotection in animal models of PD cannot be understated, given the possibility that this protection could be extended to humans [12]. However, in vivo imaging of DAT as a tool for analyzing the neuroprotective effects of DA agonists could be difficult to interpret, since DAT might be regulated by DA agonists [25,48]. Our data are consistent with this hypothesis and suggest that multiple measures of DA fiber integrity are required to assess neuroprotection by agents [49,50].

## Methods

All animals were treated in accordance with a protocol approved by the Sun Health Research Institute Animal Care and Use Committee.

### MPTP Treatment

We bred C57BL/6 mice from breeding pairs obtained from Jackson Laboratories. Eighteen male C57BL/6 mice 8–10 months of age were used for the experiments and were handled for 1 week prior to treatment. They had free access to food and water, and were maintained in a 12 h light/dark cycle prior to treatment. Mice were divided into 4 groups: the first received only vehicle (0.9% saline, 0.1 ml/10 mg body wt) (SAL-SAL), the second received pramipexole plus vehicle (PPX-SAL), the third received vehicle plus MPTP (RBI, MA) (SAL-MPTP), and the fourth received pramipexole plus MPTP (PPX-MPT). For those receiving pramipexole (Pharmacia Corporation, Kalamazoo, MI), the drug was dissolved in 0.9% sterile saline and administered by i.p. injection (0.1 ml/10 mg body wt). A single daily dose of 0.1 mg/kg body weight of pramipexole was given for 5 days. Those not receiving pramipexole were given identical injections of saline., Following pramipexole or vehicle injections on days 4 and 5, mice were given injections of either MPTP (20 mg/kg; s.c.) or vehicle (saline) twice daily at 8 h intervals.

After 7-day recovery period following the last injection of MPTP or vehicle, animals were euthanized by intracardiac perfusion with 4% paraformaldehyde in 0.15 M phosphate buffer (pH 7.2) following overdose with pentobarbital 120 mg/kg body weight i.p. Brains were removed, postfixed in the perfusion fixative for 24 h at 4°C and transferred to 30% sucrose solution for additional 24 h incubation at 4°C. The tissue was frozen on dry-ice and sectioned in cryostat at 20 μm thickness. Sections were placed in cryoprotectant solution for long-term storage at -80°C.

### Neurohistopathology

Every 10th section at the level of striatum was processed for visualization of DAT and tyrosine hydroxylase (TH), and every 5^th ^that of the midbrain processed for the visualization of TH-positive cell bodies using the avidin-biotin procedure [26,51]. Immediately adjacent sections from the midbrain were stained for cresyl violet for detection of cells. The sections were washed in phosphate buffer to remove cryoprotectant, incubated with 5% goat serum for 30 min to block background staining and incubated with anti-DAT (Chemicon, CA) or anti-TH (Chemicon, CA) at 1:1000 dilution overnight at room temperature. Control sections were treated with identical solutions but with no primary antibody. Sections were rinsed and incubated with biotinylated secondary anti-rabbit antiserum at a 1:500 dilution (Vector, CA) for 90 min at room temperature. Sections were again rinsed, incubated in streptavidin-peroxidase complex (Vector, CA) at a 1:250 dilution for 2 h at room temperature. After more thorough rinsing, sections were processed for DAB with nickel enhancement. Sections were then rinsed in phosphate buffer, mounted on gelatin-coated slides, air-dried, dehydrated, and cleared in xylene and mounted with Permount.

Unbiased stereological quantification of Nissl-stained and TH-IR neurons in the SNpc and VTA was used to estimate cell number in the midbrain. The general routine at low magnification involved use of a sampling grid for the SNpc and VTA. At high magnification the computer-based imaging system randomly selected a region of the grid and clearly definable neurons was counted within the 3-dimensional block. This was repeated for every 10^th ^region of each section. Estimation of total neuron number was based upon actual cell counts, tissue thickness, total area of designated region, and the total number of sections analyzed per animal. Group means and variances were calculated.

Our routine quantitative measurement of the optical density of regions of the striatum stained for TH-IR and DAT-IR [26,51] was employed. Using a Macintosh-based image analysis system with CCD camera and imaging software (BRAIN version 3.0, Drexel University) optical density measurements calibrated to an external standard (Kodak density step tablet) of the region of interest (ROI) and a control region (corpus callosum) of each section were made, the ratio of the ROI to control region was calculated, and the average for each animal determined. Group means and variances were estimated. Statistical analysis of group differences were assessed by ANOVA with pairwise comparisons performed using post-hoc t-tests and the Bonferroni correction.

### Testing of whether pramipexole administration alters dopamine transporter function

Fourteen C57/Bl6 mice (25–30 g, 6 months) were divided into 2 groups: one group received pramipexole (0.1 mg/kg) once a day for 5 days and the other group received the vehicle (saline). An additional 10 WT and 10 D_3 _receptor knockout mice (25–30 g, 6 months), bred according to our previous methods [30], were treated similarly. Twenty-four hours after the last injection of pramipexole or vehicle the mice were euthanized using CO_2 _narcosis and the brains rapidly removed and snap-frozen in liquid nitrogen. *K*_m _and *V*_max _values for [^3^H]1-methyl-4-phenylpyridinium (MPP+) and [^3^H]DA uptake in synaptosomes derived from the striatum were deterimined by the method of Eshleman et al [28], with minor modifications. Comparison of mean values for the *K*_m _and *V*_max _of [^3^H]MPP+ and DA uptake were made by t-test (0.05 level of significance).

### [^3^H]MPP+ and [^3^H]dopamine uptake in mouse striatal postnuclear preparations

Mouse striata were dissected and homogenized with a glass-Teflon homogenizer in ice-cold modified HEPES (1 ml). The sample was centrifuged at 1000 g, for 10 min at 4°C. The supernatant was collected and centrifuged at 14,000 g for 10 min at 4°C. The pellet was resuspended in 8 ml of HEPES buffer (HEPES 25 mM, NaCl 122 mM, CaCl 2.5 mM, MgSO_4 _1.2 mM, pargyline 10 uM, glucose 0.2%, ascorbic acid 0.02%, pH7.4). To the 8 ml sample, butaclamol was added at a final concentration of 100 nM, 4 ml of sample was than removed and placed in a separate 15 ml centrifuge tube (VWR, Pennsylvania) and Mazindol (Sigma, Missouri) was added at a final concentration of 40 uM. 50μl of the samples were added to borosilicate tubes (Fisher, Texas) and placed in a 25°C water bath with the drugs for a 10 min preincubation. The assay was initiated by adding 50μl concentrations of unlabeled MPP+ or DA ranging from 0–300 nM with [^3^H]MPP+ or [^3^H]DA at a final concentration of 20 nM. The samples were incubated at 25°C for 10 min. Specific uptake was defined as the difference in uptake observed in the absence and presence of mazindol (40μM). Uptake was terminated after 5 min by filtration through Whatman GF/C filters presoaked in HEPES buffer. Scintillation fluid was added to each filtered spot and radioactivity remaining on the filters was determined using a Wallac β-scintillation spectrometer. Each experiment involved triplicate determinations, and 6 independent experiments for each drug competition curve were performed.

### Dopamine transporter (DAT) autoradiography

10 WT and 10 D_3 _receptor knockout mice (25–30 g, 6 months) were divided into 2 groups: one group received pramipexole (0.1 mg/kg) once a day for 5 days and the other group received the vehicle (0.9% saline). Twenty-four h after the last injection of pramipexole or vehicle, the mice were overdosed as above and the brains rapidly removed and frozen on dry- ice. An additional group of 10 WT and 10 D_3 _receptor knockout mice (25–30 g, 6 months) were similarly treated but their brains were processed 14 days after the last treatment. Autoradiography of DAT sites were quantified following labeling with [^125^I]RTI-55 (3ß-(4-iodophenyl)tropan-2 ß-carboxylic acid methyl ester) (Dupont, New England Nuclear, Boston, MA) in the presence of 100 nM paroxetine (Smith Klein Beecham BRL 29060A) to block the serotonin transporter, according to published methodology [30]. Specific binding was defined with 40μM benztropine (Sigma, St. Louis MO), and amounted to 95% of total binding. Sections were apposed to ^3^H-Hyperfilm for 18 h for DAT. Autoradiographs were analyzed using a computer-based image analysis system (AIS, Imaging Research Inc., Ontario Canada) that converts transmitted optical density to the amount of radioligand bound in pmol per microgram of protein.

## List of abbreviations

D_3 _KO = D_3 _receptor knockout

DA = dopamine

DAT = dopamine transporter

MPP+ = 1-methyl-4-phenylpyridinium

MPTP = 1-methyl-4-phenyl-1,2,3,6-tetrahydropyridine

PD = Parkinson's disease

SN = substantia nigra

TH = tyrosine hydroxylase

VTA = ventral tegmental area

WT = wild type

## Competing interests

The author of this manuscript received a grant from Pharmacia Corporation to partially support the costs of the research. This company is no longer in existence.

## Authors' contributions

CW and HR carried out the MPTP treatment, performed the immunocytochemistry and participated in the statistical analysis. SB and DH carried out the uptake assays, performed the DAT autoradiography and participated in the statistical analysis. JNJ conceived of the study, and participated in its design and coordination. All authors read and approved the final manuscript.

## Figures and Tables

**Table 1:** Effects of genotype and drug on [^3^H]DA uptake and DAT density

			**[^3^H]DA uptake**		**DAT density**		
**Group**	**N**	**Vmax**	**SD**	**Km**	**SD**	**DAT**	**SD**

WT w Veh	4	516.1	± 56.5	56.7	± 18.6	10.6	± 2.5
D3 KO w Veh	4	212.4	± 18.2	10.8	± 5.7	10.8	± 1.5
WT w PPX	6	183.4	± 34.3	28.4	± 18.5	9.9	± 3.0
D3 KO w PPX	6	223.6	± 36.4	10.4	± 10.9	9.4	± 2.6

Wild-type and D_3 _KO mice were administered vehicle (0.9% saline) or pramipexole (PPX, 0.1 mg/kg I.P.) once a day for 5 days. Twenty-four hours after the last injection the brains were removed, the CPu dissected and fresh frozen ([^3^H]DA uptake) or brains frozen on dry-ice ([^125^I] RTI-55 autoradiography).

[^3^H]DA uptake Vmax reported as pmol/mg P/5 min and Km reported as nM. [^125^I] RTI-55 autoradiography for DAT density reported as fmol/μg P.
